# Supplementary material for: Dynamic interplay of microtubule and actomyosin forces drive tissue extension
Source: Nat Commun. 2024 Apr 12;15:3198. doi: 10.1038/s41467-024-47596-8 (PMC11014958; doi:10.1038/s41467-024-47596-8)
Supplement: Supplementary file 1 — Supplementary Information [file 41467_2024_47596_MOESM1_ESM.pdf]

Supplementary Information

Dynamic Interplay of Microtubule and Actomyosin Forces Drive Tissue Extension

Amrita Singh[1, 2, 5], Sameedha Thale[1, 2, 5], Tobias Leibner[3], Lucas Lamparter[2, 4], Andrea Ricker[4], Harald Nüsse[4], Jürgen Klingauf[2, 4],Milos Galic[2, 4], Mario Ohlberger[3] and Maja Matis[1, 2, 4, *]

Affiliations

[1]Institute of Cell Biology, Medical Faculty, University of Münster, Münster, Germany.

[2]'Cells in Motion' Interfaculty Centre, University of Münster, Münster, Germany.

[3]Applied Mathematics, Institute for Analysis and Numerics, University of Münster, Münster, Germany.

[4]Institute of Medical Physics and Biophysics, Medical Faculty, University of Münster, Münster, Germany.

[5]These authors contributed equally.

* To whom correspondence should be addressed: matism@uni-muenster.de

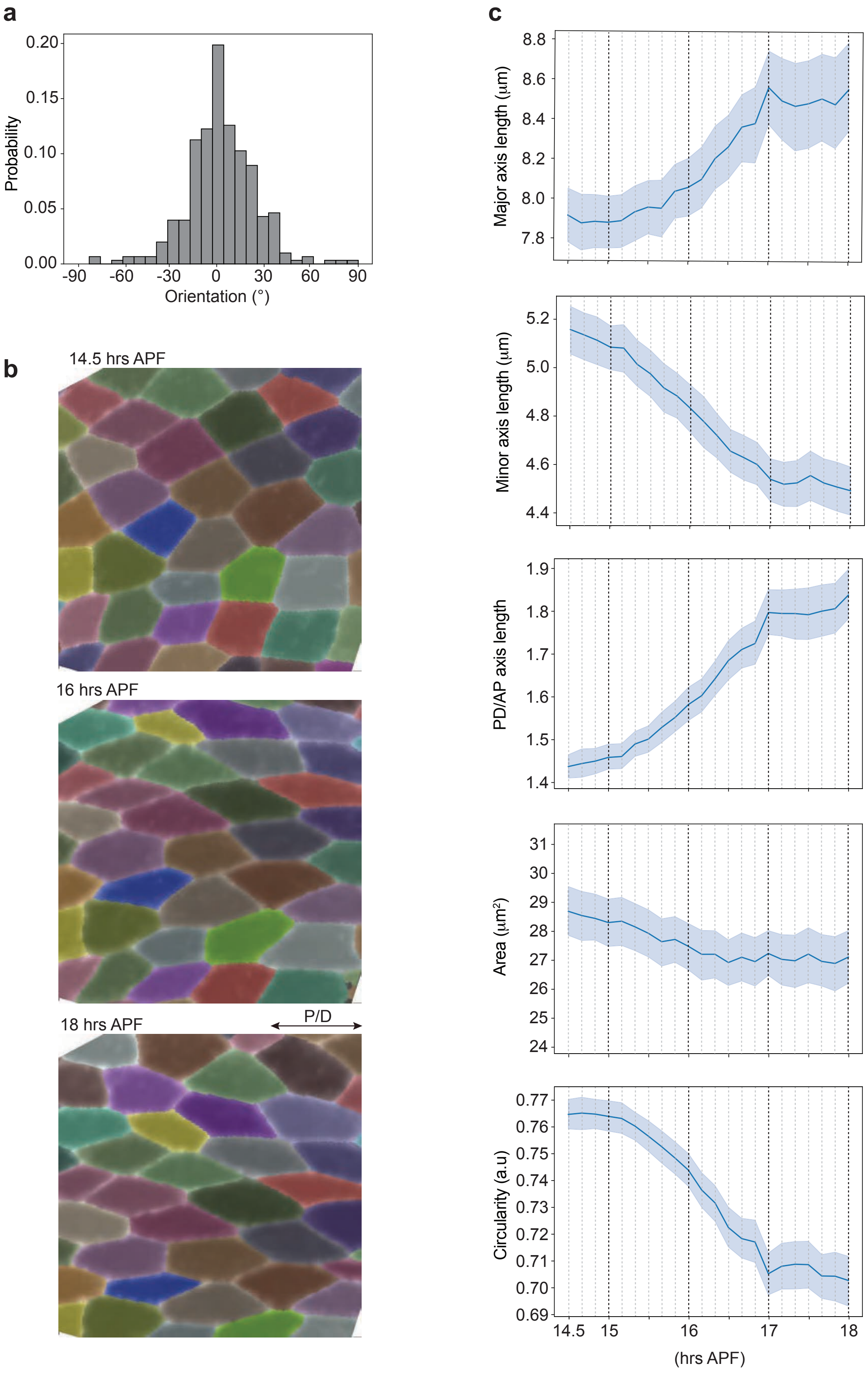

Singh et al.
Supp. Figure 1

Supplementary Figure 1. Collective cell elongation drives initial wing elongation (a) Graph indicating the axis of cell elongation during wing development (-30° to 30° is along the P/D axis). (b) Representative images from live imaging of developing wing at different time points marked by Arm-GFP. Movies of developing wings are tracked and color-coded with an automated script for analyzing cell shape parameters. (c) Graphs indicate the evolution of the cell's major axis, minor axis, PD/AP axis length (cell elongation), area, and circularity in the developing wing. The images shown are representative of 3 independent movies. Source data are provided as a Source Data file.

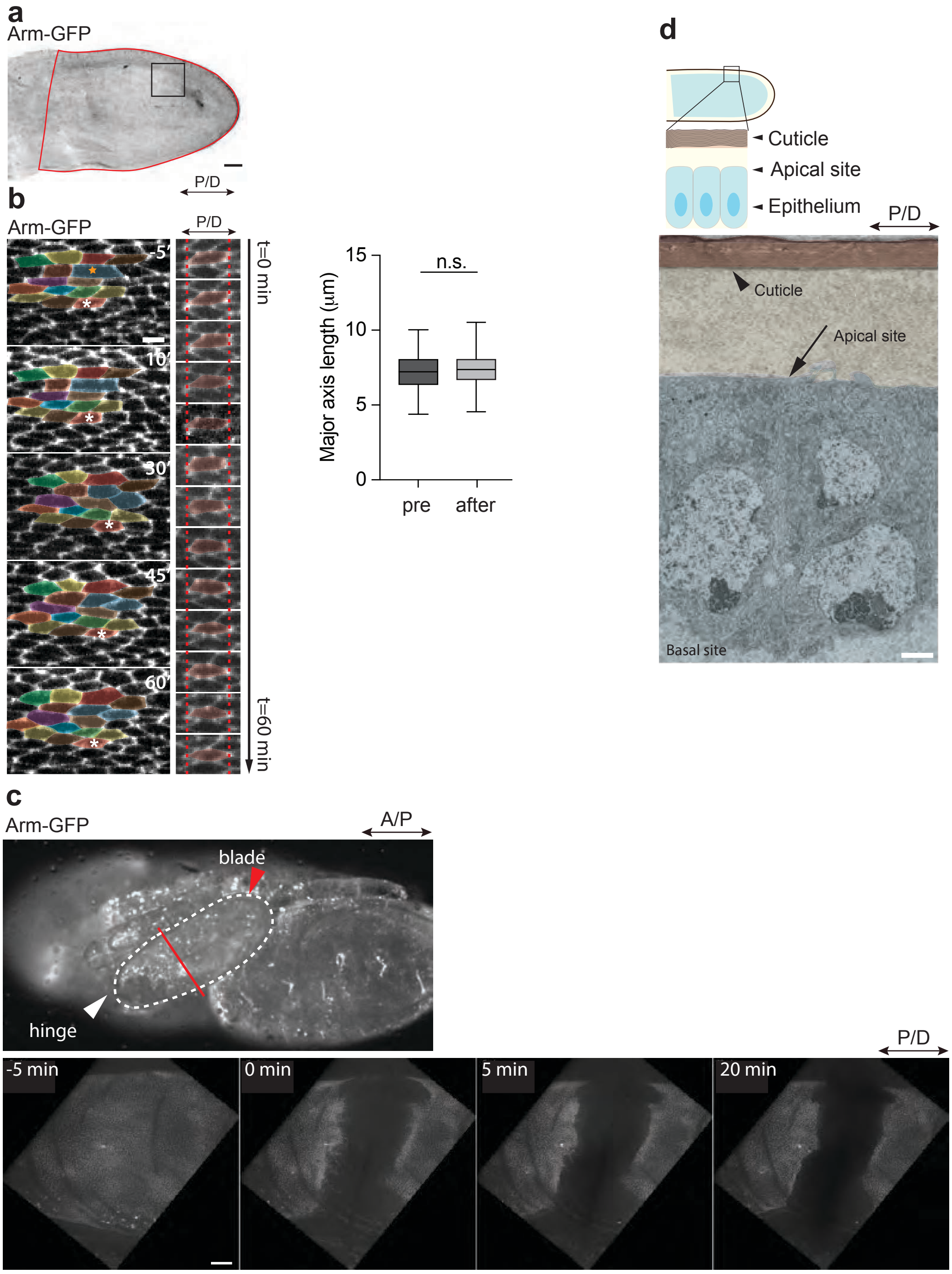

Singh et al.
Supp. Figure 2

Supplementary Figure 2. Initial wing cell elongation is independent of extrinsic forces

(a) Representative image of 18 hAPF control (*arm-Arm-GFP*) wing marked by a red line indicating the area used for wing length quantification and a black box marking the ROI used for cell parameter measurements. (b) Representative images of 18 hAPF wings expressing Arm-GFP 5 minutes before and after hinge cut (10, 30, 45, and 60 minutes). The orange star marks the cell that will divide. Quantification of cell length in 18 hAPF wings before hinge cut and after 60 minutes (two-tailed t-test, n.s. $p > 0.1683$, n(precut)=136 and n(after cut)=125 cells and 3-5 pupae per genotype). Boxes in the plot extend from the 25th to the 75th percentiles, with a line at the median. Whiskers show min and max values. (c) Hinge ablation experiment (see Supplementary Video 1), showing whole pupae expressing Arm-GFP (top). The white dashed line marks the wing edge, and the red line marks the position of the laser cut. Below are time-lapse images of Arm-GFP before (5 min), during and after ablation of the wing (5 min and 20 min). (d) Cartoon showing a cross-section of the wing 16 hAPF (top) and TEM micrograph of a cross-section of 16 hAPF control *(w^{1118})* wing (bottom). Note that the overlying cuticle (black arrowhead) is entirely detached from the epithelium apical surface (black arrow). Source data are provided as a Source Data file. Scale bars, (b) 5 μm, (c) 25 μm and (d) 1 μm.

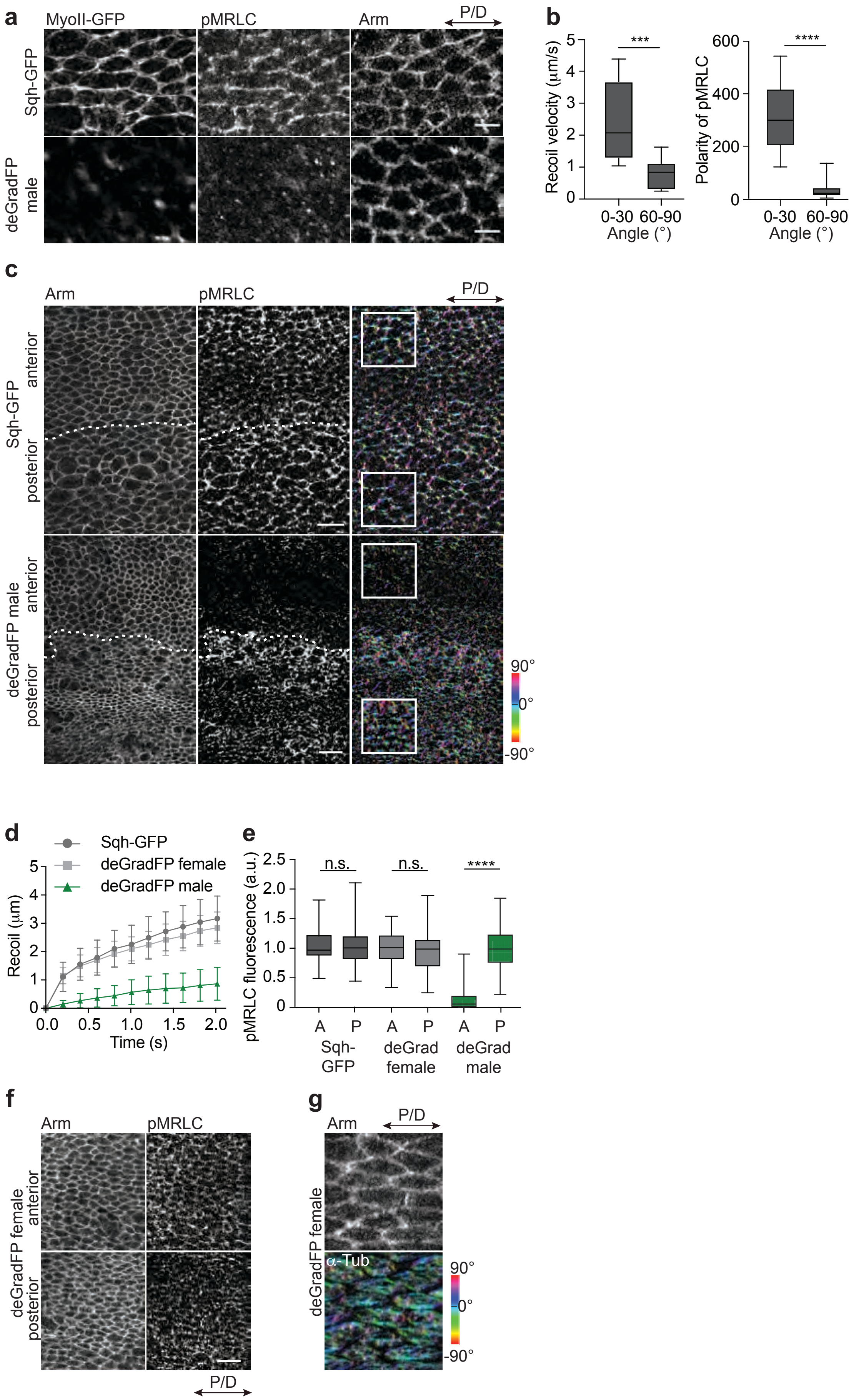

Singh et al.
Supp. Figure 3

Supplementary Figure 3. Myosin II is polarized during early pupal wing development

(a) Representative images showing the anterior compartment of Sqh-GFP (*sqh^{AX3}/Y; sqh-Sqh-GFP/tubGal80^{ts}*; ciGal4/+) male (top) and deGradFP (*sqh^{AX3}/Y; sqh-Sqh-GFP/tubGal80^{ts}*; ciGal4/*UAS-NSlmb-vhhGFP4*) male (lower). To obtain a developmental stage equivalent to 18 hAPF at 25°C, deGradFP wings were grown sequentially at 18°C and 29°C using the tub-Gal80^{ts} system (see Table S1). Wings express Sqh-GFP and are stained for pMRLC and Arm. In control flies (Sqh-GFP male), Myosin II and pMRLC show polarized distribution at junctions oriented along the P/D axis. In deGradFP-expressing flies, Sqh-GFP and the pMRLC signal are lost from junctions. The remaining Sqh-GFP signal did not colocalize with pMRLC staining. Images shown in (a) are representative of 5 wings and 3 independent experiments. (b) Quantification of recoil velocity upon laser ablation of P/D (0-30°) and A/P (30-60°) interfaces in 18 hAPF control (*arm-Arm-GFP*) flies (two-tailed t-test, *** $p = 0.0003$, n=12 junctions each and 5 pupae). Quantification of pMRLC polarity (two-tailed Mann–Whitney test, **** $p < 0.0001$, n=450 cells and 3 pupae). (c) Images showing the A/P border in control flies (Sqh-GFP male, top) and Sqh-GFP knockdown flies (deGradFP male, bottom) stained for Arm and pMRLC. In control flies, pMRLC localizes to the junctions in the anterior and posterior compartments. In the flies expressing deGradFP under *ci-Gal4* control, the pMRLC signal in the anterior compartment is lost compared to the wing's posterior site. The polarity of the pMRLC signal is color-coded using OrientationJ. (d) Quantification of displacement upon laser ablation for interfaces along the P/D axis in Sqh-GFP and deGradFP (*sqh^{AX3}/+; sqh-Sqh-GFP/tubGal80^{ts}*; ciGal4/*UAS-NSlmb-vhhGFP4*) female control flies (gray, light gray) and Sqh-GFP knockdown flies (deGradFP male, green). n =10 junctions and 4 pupae per genotype. Values on the graph show mean displacements. Error bars show sd. (e) Quantification of mean intensities of pMRLC in anterior and posterior compartments for Sqh-GFP and deGradFP female control flies (gray, light gray) and Sqh-GFP knockdown flies (deGradFP male, green). (Kruskal–Wallis test, from left to right: n.s. $p > 0.9999$, n.s. $p > 0.9999$, **** $p < 0.0001$, number of junctions for Sqh-GFP male (A)=90, (P)=77, deGradeFP female (A)=76, (P)=74 and for deGradFP male (A)=100, (P)=102 and 3-5 pupae per genotype). For (b, e), boxes in the plot extend from the 25th to the 75th percentiles, with a line at the median. Whiskers show min and max values. (f) Images showing anterior (top) and posterior (bottom) compartments in deGradFP female

control flies (having one wild-type and one mutant copy of null mutation of *sqh*^{AX3}) stained for Arm and pMRLC. In deGradFP female flies, pMRLC localizes to the junctions in the anterior and posterior compartments. (g) Images of 18 hAPF wing deGradFP female stained for Arm and α-Tub to visualize microtubules. The orientation of microtubules is color-coded using OrientationJ. The images shown are representative of 4 wings and 3 independent experiments. Source data are provided as a Source Data file. Scale bars, (a) 5 µm, (c, f) 10 µm.

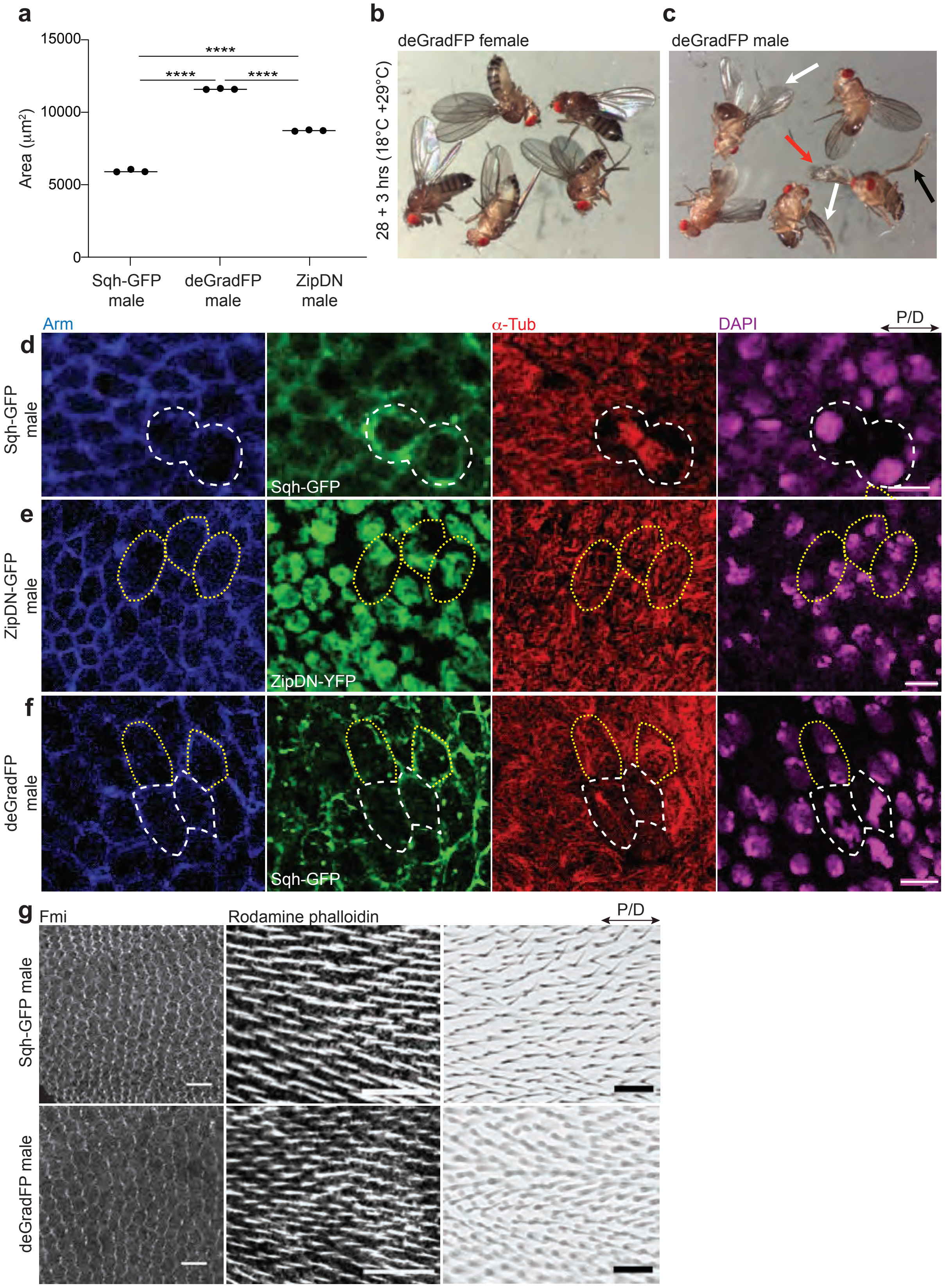

Singh et al.
Supp. Figure 4

Supplementary Figure 4. deGradFP-mediated knockdown of Sqh-GFP produces known phenotypes consistent with loss of Myosin II function in *Drosophila* pupal wing (i.e., vein expansion, blisters and aberrant cell division)

(a) Quantification of L2 vein area for control (*sqh*^{AX3}*/Y; sqh-Sqh-GFP/tubGal80*^{ts}; ciGal4/+) and Myosin II-depleted wings (deGradFP (*sqh*^{AX3}*/Y; sqh-Sqh-GFP/tubGal80*^{ts}; ciGal4/*UAS-NSlmb-vhhGFP4*) male and ZipDN-GFP (*UAS-GFP-DN-Zip/tubGal80*^{ts}; ciGal4/+) male) (Ordinary one-way ANOVA, **** $p < 0.0001$, n=3 veins and 3 pupae per genotype). The graph shows bars indicating the mean and sd. (b and c) Images showing deGradFP (*sqh*^{AX3}*/+; sqh-Sqh-GFP/tubGal80*^{ts}; ciGal4/*UAS-NSlmb-vhhGFP4*) female (b) and deGradFP male (c) adult flies grown at a combination of 18°C and 29°C temperature regimes using the tub-Gal80^{ts} system. Although adult wing hair orientation appears unaffected upon deGradFP expression (see panel g), some other defects are visible. With as little as 3 hrs of deGradFP expression, deGradFP male wings (c) show defects such as blisters (red arrows), curled up (black arrows) and droopy wings (white arrows). In contrast, deGradFP female (b) adult flies raised under the same conditions do not show any obvious wing phenotype(s) and can fly normally. Images shown in (b, c) are representative of 10 adult flies and 3 independent experiments. (d-f) Images showing cell division event(s) in control flies Sqh-GFP male (d), ZipDN-GFP male (e) and deGradFP male (f). Cells express Sqh-GFP (d,f) and ZipDN-GFP (e) stained with anti-Arm antibody to visualize cell outlines apically, anti-α-Tub antibody to visualize microtubules and DAPI to visualize nuclei in the cells. Cells in Sqh-GFP male (d) have two nuclei only when the cells undergo division, as evident by the presence of spindle microtubules, Myosin II enrichment at cleavage sites and rounded-up cell morphology (all indicated by broken white lines within the corresponding panels). However, cells in ZipDN-GFP male (e) and deGradFP male (f) show two nuclei (yellow dotted lines) even when the cells are not undergoing division, as evident by the absence of spindle microtubules and presence of apical planar microtubules (yellow dotted lines) and nonrounded or elongated cell morphology (yellow dotted lines) when compared to cells undergoing division where spindle microtubules (white broken lines) and nuclear division (white broken lines) are observed. Images shown in (d-f) are representative of n=4 pupae and N=3 independent experiments. (g) Representative images of Sqh-GFP male and deGradFP male wigs equivalent to 24 hAPF (left panels) and 30 hAPF (middle panels)

at 25°C and in adult animals show that wing PCP is independent of MyosinII activity. Wings were stained with anti-Flamingo antibody to visualize PCP organization and rhodamine-phalloidin to visualize prehair orientation. Images shown are representative of n=6 wings and N=3 independent experiments. Source data are provided as a Source Data file. Scale bars, (d-f) 5 μm, (g, left) 10 μm, (g, middle, right) 20 μm.

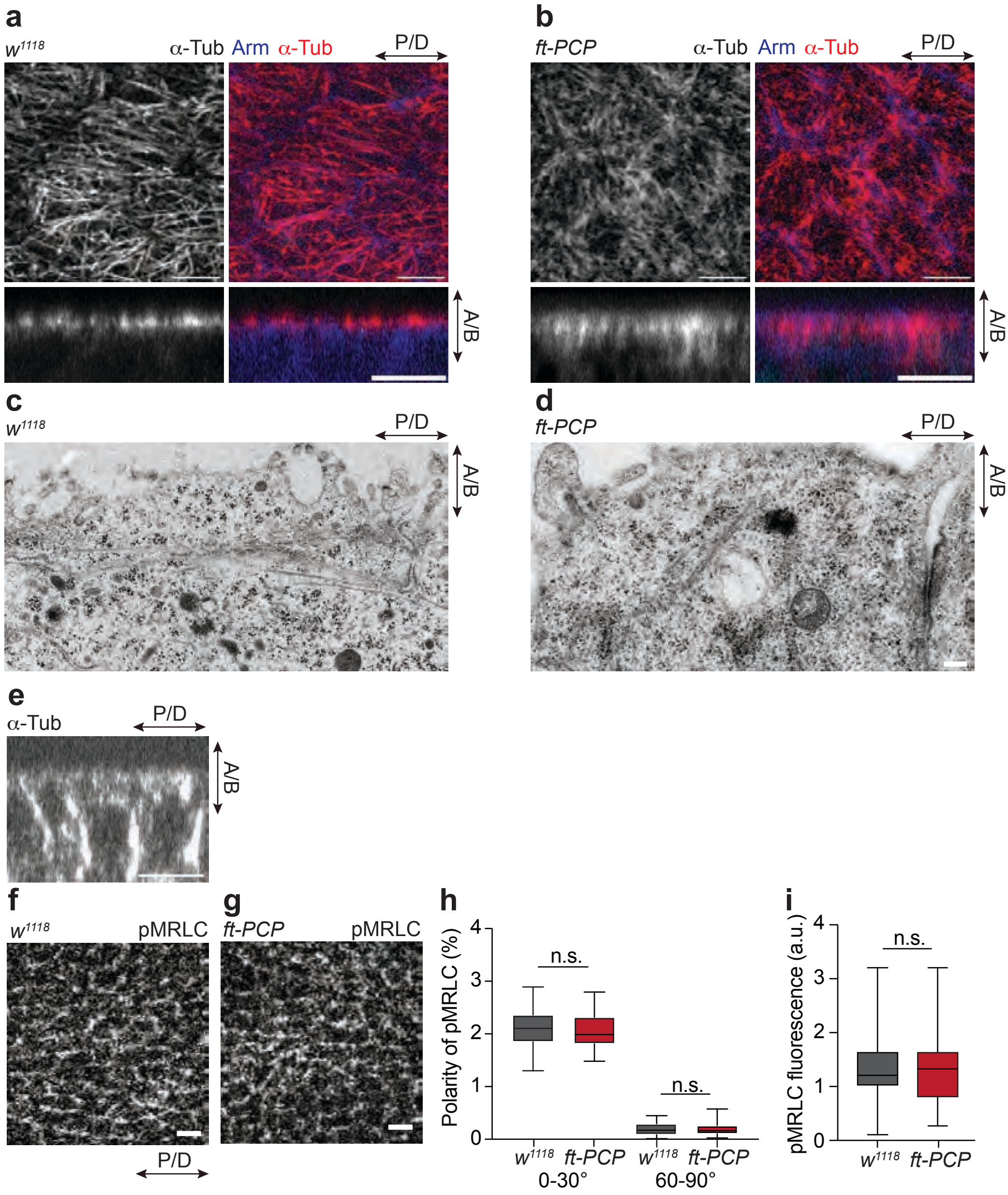

Singh et al.
Supp. Figure 5

Supplementary Figure 5. The Fat-PCP pathway regulates the organization of non-centrosomal microtubules within the plane and does not affect actomyosin (a, b) Representative images of 18 hAPF control *(w^{1118})* (a) and *ft-PCP* (*$ft^{l(2)\,fd}$/ft^{GRV};act-Gal4/UAS-FtΔECDΔN-1*) (b) mutant stained for Arm and α-Tub to visualize microtubules. Orthogonal views (lower panel) indicate that remodeling from lateral to planar polarized microtubules is impaired in the *ft-PCP* mutant. The images shown are representative of 5 wings and 3 independent experiments. (c, d) TEM micrograph showing a cross-section of control (c) and *ft-PCP* mutant (d) wings. (e) Orthogonal view of the *ft-PCP* mutant wing at an early stage of development showing lateral bundles of microtubules. (f, g) Representative images of 18 hAPF control (f) and *ft-PCP* mutant (g) stained for pMRLC. (h) Quantification of pMRLC polarity in control and *ft-PCP* wings using Orientation J (Kruskal–Wallis test, (0-30°) n.s. $p = 0.9040$, (60-90°) n.s. $p > 0.9999$, n=320 cells and 3 pupae). (i) Quantifications of mean intensities of pMRLC in control and *ft-PCP* wings (Mann–Whitney test, n.s. $p > 0.5360$, n(*w^{1118}*)=120 and n(*ft-PCP*)=94 junctions and 3-5 pupae per genotype). Boxes in all plots extend from the 25th to the 75th percentiles, with a line at the median. Whiskers show min and max values. Source data are provided as a Source Data file. Scale bars, (a, b, e-g) 5 µm and (c, d) 200 nm.

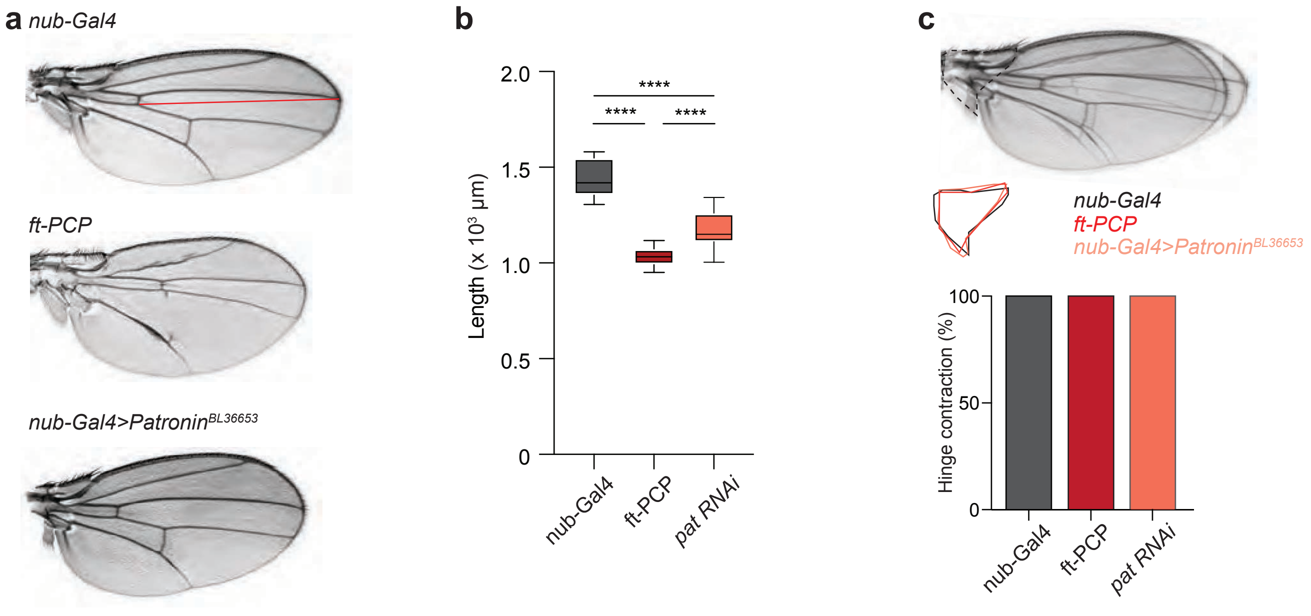

Singh et al.
Supp. Figure 6

Supplementary Figure 6. In the *ft-PCP* mutant and Patronin-depleted wings, the hinge contracts normally

(a) Representative images of control (*nub-Gal4*), *ft-PCP,* and Patronin-depleted (*nub-Gal4>Patronin*^{RNAi}) adult wings. Patronin depletion does not affect apoptosis (see Supplementary Fig. 7). Thus, it does not affect wing shape. (b) Graph showing quantification of wing lengths as marked by the red line in (a) (Kruskal–Wallis test: **** $p < 0.0001$, **** $p < 0.0001$ and **** $p < 0.0001$, N(*nub-Gal4*)=99, n(*ft-PCP*)=29 and n(*nub-Gal4>Patronin*^{RNAi})=79 wings). Boxes in the plot extend from the 25th to the 75th percentiles, with a line at the median. Whiskers show min and max values. (c) Overlay of adult wings from different genotypes indicating no defects in hinge contraction. The middle panel shows the quantification of hinge contraction in control, *ft-PCP*, and Patronin-depleted wings. In control flies, 39/39 wings display normal hinge contraction; in *ft-PCP* adults, 47/47 wings; and in Patronin-depleted adults, 52/52 wings. Source data are provided as a Source Data file.

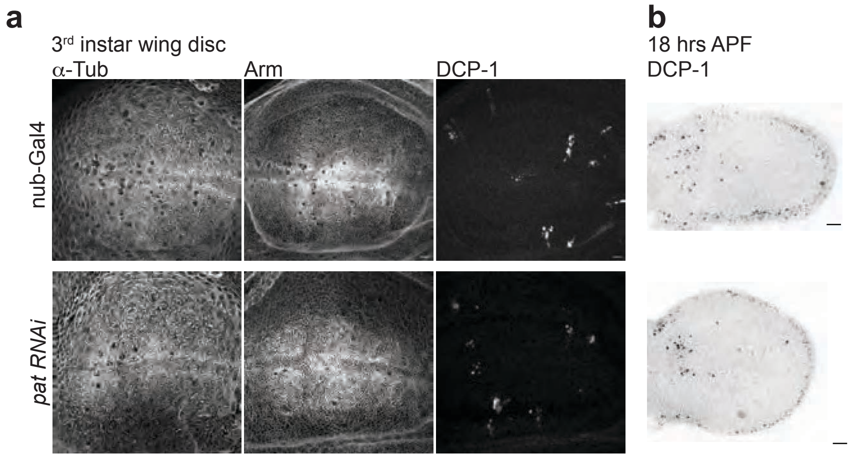

Singh et al.
Supp. Figure 7

Supplementary Figure 7. Patronin shows no effect on cell apoptosis

(a) Representative images of control (*nub-Gal4*) and Patronin-depleted (*nub-Gal4>Patronin*^{RNAi}) third instar wing imaginal discs stained for α-Tub, Arm, and Dcp-1 showing no effect on cell apoptosis. The images shown are representative of 13 wing discs and 3 independent experiments. (b) Representative images of control and Patronin-depleted 18 hAPF wings stained for Dcp-1 show the same spatial apoptosis pattern only at the wing margin. The images shown are representative of 8 wings and 3 independent experiments. Scale bars, (b) 50 μm.

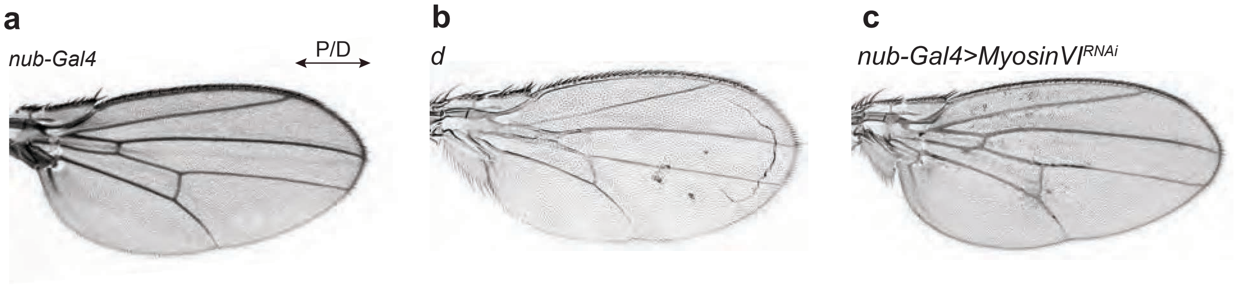

Singh et al.
Supp. Figure 8

Supplementary Figure 8. Inhibition of Dachs and Myosin VI function does not affect wing shape

(a-c) Representative images of control (w^{1118}) (a), *dachs* mutant (d^{1}/d^{GC13}) (b), and *MyosinVI* (*nub-Gal4*>*MyosinVI*^{RNAi})-depleted (c) wings showed no defects in tissue elongation. Number of analyzed wings n(*wt*)=16/16, n(*d*)= 40/40 and n(*MyosinVI*^{RNAi})=14. However, *dachs* mutant wings display abnormal vein patterning, and the knockdown of Myosin VI causes wing blisters.

Supplementary Table 1. A table summarizes the dual temperature regimes in experiments using the deGradFP system

Temperature regimes (at 18°C+29°C)	Equivalent stage (at 25°C)	Assay(s) done using the regime	Explanation of the regime (in brief)
22+6.5 hrs	18 hAPF	• Standardization for 18 hAPF pupal wing.<br>• Tension analysis (laser ablation).<br>• Cell shape and area analysis.<br>• MT alignment assay.	0 hAPF prepupae collected and grown at 18°C for 22 hrs followed by incubation at 29°C for 6.5 hrs.
22+14 hrs	24 hAPF	• PCP organization and cell area analysis<br>• Vein area analysis	0 hAPF prepupae collected and grown at 18°C for 22 hrs followed by incubation at 29°C for 14 hrs.
22+20 hrs	30 hAPF	• Pupal wing hair polarity	0 hAPF prepupae collected and grown at 18°C for 22 hrs followed by incubation at 29°C for 20 hrs.
28+3 hrs	N/A	• Adult wing hair polarity.<br>• Adult wing morphology.	0 hAPF prepupae collected and grown at 18°C for 22 hrs followed by incubation at 29°C for 3 hrs.
0+24 hrs	N/A	• Cell division defects.	0 hAPF prepupae collected and grown at 29°C for 24 hrs.
