# Supplementary material for: Dynamic interplay of microtubule and actomyosin forces drive tissue extension
Source: Nat Commun. 2024 Apr 12;15:3198. doi: 10.1038/s41467-024-47596-8 (PMC11014958; doi:10.1038/s41467-024-47596-8)
Supplement: Supplementary file 3 — Description of Additional Supplementary Files [file 41467_2024_47596_MOESM3_ESM.pdf]

Description of Additional Supplementary Files

File Name: Supplementary Movie 1
Description: Live imaging of the hinge region of 18 hAPF pupal wings expressing Arm-GFP before and after ablation. Scale bar, 25 µm.

File Name: Supplementary Movie 2
Description: 3D view of 14.5 hAPF wing expressing EOS-Tub to visualize microtubule bundles. Scale bar, 3 µm.

File Name: Supplementary Movie 3
Description: 3D view of 18 hAPF control (w1118) wing stained for α-Tub to visualize planar polarized microtubule organization. Scale bar, 5 µm.

File Name: Supplementary Movie 4
Description: 3D view of 18 hAPF ft-PCP (ftl(2) fd/ftGRV;act-Gal4/UAS-FtΔECDΔN-1) mutant wing stained for α-Tub to visualize microtubule organization. Scale bar, 5 µm.

File Name: Supplementary Movie 5
Description: 3D view of 18 hAPF Patronin-depleted (nub-Gal4>PatroninRNAi) wing stained for α-Tub to visualize microtubule organization. Scale bar, 5 µm.
