# Supplementary material for: Dynamic interplay of microtubule and actomyosin forces drive tissue extension
Source: Nat Commun. 2024 Apr 12;15:3198. doi: 10.1038/s41467-024-47596-8 (PMC11014958; doi:10.1038/s41467-024-47596-8)
Supplement: Supplementary file 9 — Reporting Summary [file 41467_2024_47596_MOESM9_ESM.pdf]

nature portfolio

Corresponding author(s): Maja Matis

Last updated by author(s): Mar 26, 2024

Reporting Summary

Nature Portfolio wishes to improve the reproducibility of the work that we publish. This form provides structure for consistency and transparency in reporting. For further information on Nature Portfolio policies, see our Editorial Policies and the Editorial Policy Checklist.

Statistics

For all statistical analyses, confirm that the following items are present in the figure legend, table legend, main text, or Methods section.

n/a	Confirmed	
☐	☒	The exact sample size (*n*) for each experimental group/condition, given as a discrete number and unit of measurement
☐	☒	A statement on whether measurements were taken from distinct samples or whether the same sample was measured repeatedly
☐	☒	The statistical test(s) used AND whether they are one- or two-sided<br>*Only common tests should be described solely by name; describe more complex techniques in the Methods section.*
☒	☐	A description of all covariates tested
☐	☒	A description of any assumptions or corrections, such as tests of normality and adjustment for multiple comparisons
☐	☒	A full description of the statistical parameters including central tendency (e.g. means) or other basic estimates (e.g. regression coefficient) AND variation (e.g. standard deviation) or associated estimates of uncertainty (e.g. confidence intervals)
☐	☒	For null hypothesis testing, the test statistic (e.g. *F*, *t*, *r*) with confidence intervals, effect sizes, degrees of freedom and *P* value noted<br>*Give P values as exact values whenever suitable.*
☒	☐	For Bayesian analysis, information on the choice of priors and Markov chain Monte Carlo settings
☒	☐	For hierarchical and complex designs, identification of the appropriate level for tests and full reporting of outcomes
☒	☐	Estimates of effect sizes (e.g. Cohen's *d*, Pearson's *r*), indicating how they were calculated

Our web collection on statistics for biologists contains articles on many of the points above.

Software and code

Policy information about availability of computer code

Data collection	VisiView software and ZEN software were used for image aquisition
Data analysis	All statistical tests were made in Prism9 (GraphPad Software, La Jolla, CA, USA). All images were processed using Fiji freeware software (v 2.14.0/1.54)and custom-written programs in Jupyter Notebook (Jupyter.org)

For manuscripts utilizing custom algorithms or software that are central to the research but not yet described in published literature, software must be made available to editors and reviewers. We strongly encourage code deposition in a community repository (e.g. GitHub). See the Nature Portfolio guidelines for submitting code & software for further information.

Data

Policy information about availability of data

All manuscripts must include a data availability statement. This statement should provide the following information, where applicable:

- Accession codes, unique identifiers, or web links for publicly available datasets
- A description of any restrictions on data availability
- For clinical datasets or third party data, please ensure that the statement adheres to our policy

All data are available in the main text or the supplementary materials.

Research involving human participants, their data, or biological material

Policy information about studies with human participants or human data. See also policy information about sex, gender (identity/presentation), and sexual orientation and race, ethnicity and racism.

Reporting on sex and gender	No human studies were performed
Reporting on race, ethnicity, or other socially relevant groupings	No human studies were performed
Population characteristics	No human studies were performed
Recruitment	No human studies were performed
Ethics oversight	No human studies were performed

Note that full information on the approval of the study protocol must also be provided in the manuscript.

Field-specific reporting

Please select the one below that is the best fit for your research. If you are not sure, read the appropriate sections before making your selection.

[x] Life sciences [ ] Behavioural & social sciences [ ] Ecological, evolutionary & environmental sciences

For a reference copy of the document with all sections, see nature.com/documents/nr-reporting-summary-flat.pdf

Life sciences study design

All studies must disclose on these points even when the disclosure is negative.

Sample size	The sample sizes were chosen empirically based on the observed effects (listed in the figure legends) and following general guidelines.
Data exclusions	No data were excluded
Replication	All attempts at replication were successful. All experiments presented in the manuscript were repeated at least in three independent experiments/biological replicates.
Randomization	The flies of the same genotypes were collected together and been randomly allocated into different groups.
Blinding	The experiments were not performed blinded, as the genotypes of the fly strains had to be visible so that appropriate treatment could be carried out or stained with appropriate antibodies.

Reporting for specific materials, systems and methods

We require information from authors about some types of materials, experimental systems and methods used in many studies. Here, indicate whether each material, system or method listed is relevant to your study. If you are not sure if a list item applies to your research, read the appropriate section before selecting a response.

Materials & experimental systems

n/a	Involved in the study	
[ ]	[x]	Antibodies
[x]	[ ]	Eukaryotic cell lines
[x]	[ ]	Palaeontology and archaeology
[ ]	[x]	Animals and other organisms
[x]	[ ]	Clinical data
[x]	[ ]	Dual use research of concern
[x]	[ ]	Plants

Methods

n/a	Involved in the study	
[x]	[ ]	ChIP-seq
[x]	[ ]	Flow cytometry
[x]	[ ]	MRI-based neuroimaging

Antibodies

Antibodies used	rabbit anti-α-Tubulin (1:200; ab18251, Abcam)<br>mouse anti-Armadillo (1:100; N2 7A1, DSHB)<br>mouse anti-Flamingo (1:100; DSHB)<br>rabbit anti-pMRLC (1:50; 3671S, Cell Signaling Technology)

	rabbit anti-Dcp-1 (1:100; #9578, Cell Signaling Technology)<br>rat anti-Tyr-Tubulin (1:750; ab6160, Abcam)<br>mouse anti- α-Tubulin (1:750; T9026, Sigma)<br>Rhodamine phalloidin dye (1:100; Invitrogen)<br>Fluorophore-conjugated secondary antibodies (Invitrogen) were used at 1:200 dilution. Secondary antibodies used in this study include Alexa Fluor 488-, 568- or 633-conjugated goat anti-rabbit, anti-mouse, anti-rat IgGs (A-11036, A-21070, A-21052, A-11006, A-11077). For nuclei staining, mouting media with DAPI was used (Vectashield Antifade mouting medium with DAPI, H-1200-10).
Validation	We used only common antibodies that are commercially available and were previously published by other labs and. The antibodies were validated based on the reported patterns.

Animals and other research organisms

Policy information about studies involving animals; ARRIVE guidelines recommended for reporting animal research, and Sex and Gender in Research

Laboratory animals	All experiments examined Drosophila melanogaster at the 3rd larval stage, pupal stage and adults. Genotypes used in each experiment/figure are detailed in legend and method section attached to the manuscript.
Wild animals	The study did not involve wild animals.
Reporting on sex	All experiments were conducted on male and female Drosophila.
Field-collected samples	No Field-collected animals were used in the study
Ethics oversight	No ethics approval or oversight is required for studies using Drosophila.

Note that full information on the approval of the study protocol must also be provided in the manuscript.

Plants

Seed stocks	The study did not involve plants.
Novel plant genotypes	The study did not involve plants.
Authentication	The study did not involve plants.
